# Improving Trainee Doctors' Awareness on How to Refer for Routine Radiological and Cardiac Investigations at a Psychiatric Hospital in South London

**DOI:** 10.1192/bjo.2024.341

**Published:** 2024-08-01

**Authors:** Annabel Felton, Trisha Ang

**Affiliations:** South London and Maudsley NHS Trust, London, United Kingdom

## Abstract

**Aims:**

Psychiatric inpatient hospital, although part of secondary care, is separate from a physical health hospital and therefore does not have access to electronic referral systems, which increases efficiency of referral processes. As part of an admission clerking for all inpatients in psychiatric hospitals, the admitting doctor takes a history of past medical issues, a physical examination, electrocardiogram and bloods. Depending on findings, further radiological and cardiac investigations may be warranted. Not having access to electronic referral systems can cause delay in delivering treatment for psychiatric inpatients, especially when referral pathways is unclear. The aim of this quality improvement project is to increase the knowledge of referrers in order to improve efficiency completing referrals and reduce incorrect referrals. With clinicians able to refer for routine imaging correctly and in an efficient manner, it is hoped that this will correlate with an improved quality of care received by patients.

**Methods:**

Firstly we assessed the knowledge of currently employed trainee doctors, via a web-based survey, on how to refer for routine and commonly ordered radiological and cardiac investigations. Employed referrers included core trainee, GP and foundation year trainee doctors. We then created an electronic referral pack which includes a guidance and referral forms provided to clinicians when they start employment at Lambeth hospital and accessible to current trainees. A follow up survey then reassessed the knowledge of these referrers.

**Results:**

There was a total of 11 responses received from survey prior to sending out the electronic referral guidance pack, of which 100% believed that it would be helpful to have a referral guidance pack. A total of 4 responses were received after sending out the guidance. The surveys showed that there is improved knowledge of how to refer for routine radiological and cardiac investigations after guidance was sent. Prior to sending the guidance, 9.1% referrers were made aware of the referral process, and this increased to 50% after the referral guidance pack was sent out.

**Conclusion:**

Trainee doctors in psychiatric hospitals require more support with physical health management in psychiatric hospitals, including referring for physical health investigations, as referrers cannot access electronic referral systems used in physical health hospitals. Results need to be correlated with clinical outcomes in future. A longer term project could include linking the electronic referral systems between psychiatric and physical health hospitals.

